# A Case of Acute Encephalitis in COVID-19 Patient: A Rare Complication

**DOI:** 10.7759/cureus.15636

**Published:** 2021-06-14

**Authors:** Shah T Sarmast, Alaa S Mohamed, Zain Amar, Sobia Sarwar, Zahoor Ahmed

**Affiliations:** 1 Neurology, California Institute of Behavioral Neurosciences and Psychology, Fairfield, USA; 2 Neurology, Augusta University, Augusta, USA; 3 Medicine, Isra University Hospital Hyderabad, Hyderabad, PAK; 4 Neurology, Independent Medical College, Faisalabad, PAK; 5 Internal Medicine, Mayo Hospital, Lahore, PAK

**Keywords:** encephalopathy, acute encephalitis, covid-19, sars-cov-2, covid pneumonia

## Abstract

Coronavirus disease 19 (COVID-19) is a respiratory disease, has a variable presentation, and neurological involvement in COVID-19 is not widely reported. We report a rare case of acute encephalitis in a COVID-19 patient presented with fever, dry cough, and dyspnea. She had a fever, tachypnea, and tachycardia. On auscultation, she had scattered wheezing in both lung fiends. Chest X-ray revealed small infiltrates in the lower lobe of both lungs. A nasopharyngeal swab for the COVID-19 polymerase chain reaction was positive. Later on, she developed sudden onset confusion accompanied by restlessness and visual hallucinations. Neurological examination revealed an altered level of consciousness, slight trembling of the limbs, psychomotor restlessness, and poor speech with no signs of meningeal irritation. Magnetic resonance imaging of the brain revealed diffuse hyperintense signals. A possible diagnosis of acute encephalitis was made due to concurrent COVID-19 infection and lack of other findings suggesting a diagnosis other than COVID-19. She was treated with azithromycin, tocilizumab, and methylprednisolone. Her condition started improving gradually.

## Introduction

Coronavirus disease 19 (COVID-19), caused by the severe acute respiratory syndrome coronavirus 2 (SARS-CoV-2), is a challenging concern for physicians due to its variable presentation [1]. COVID-19 is a respiratory disease and has a broad spectrum of clinical manifestations and clinical syndromes ranging from subclinical infection to severe infection such as acute respiratory distress syndrome (ARDS) and lung failure [2]. In addition, the novel coronavirus has also been associated with severe metabolic syndromes, acute kidney injury, thromboembolic events, acute pancreatitis, cardiovascular events, and neurological syndromes, including acute cerebrovascular disease and encephalopathy [3-7]. Neurological manifestations can be mild, leading to headache and dizziness or, more critically, causing encephalitis, encephalomyelitis, and stroke [8,9]. However, acute encephalitis due to COVID-19 is not widely reported, and data on COVID-19-induced encephalitis are lacking. Herein, we describe a case of acute encephalitis caused by SARS-CoV-2.

## Case presentation

**Figure 1:**
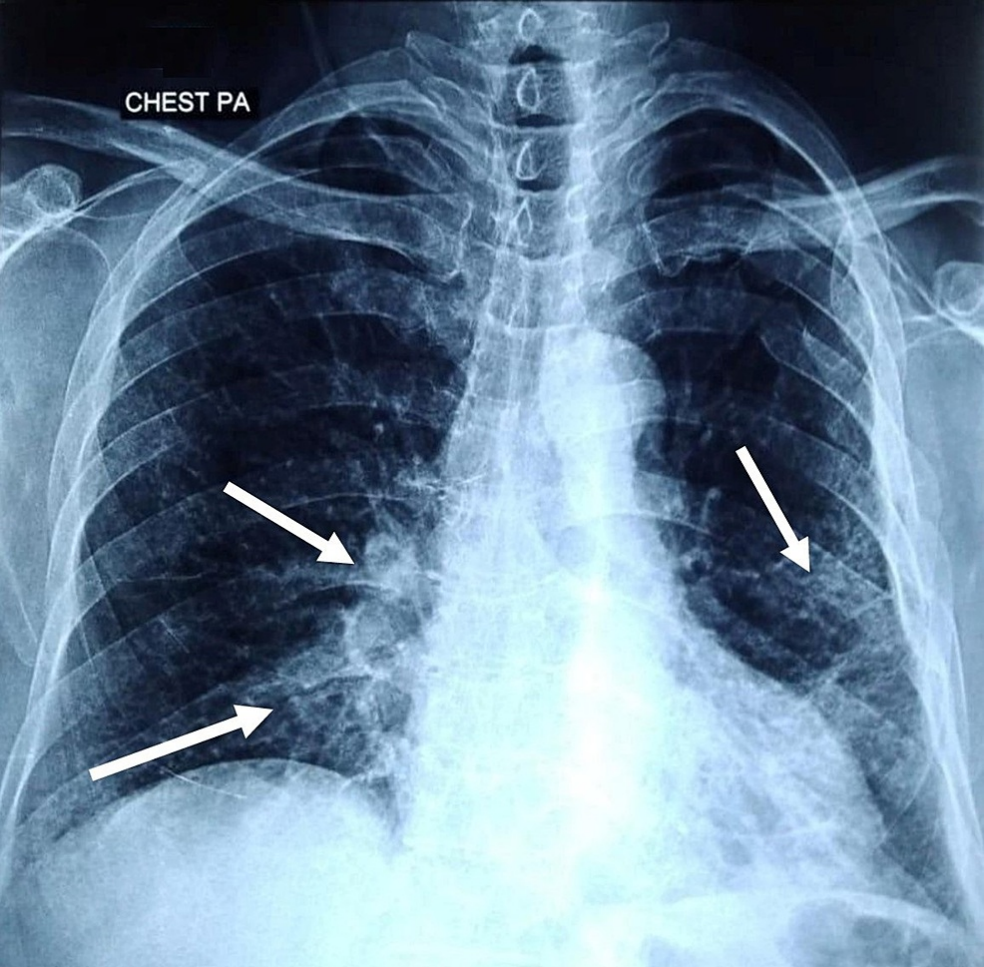
Chest X-ray showing infiltrates in the lower lobe of both lungs (white arrows).

A 63-year-old female with a past medical history of hypothyroidism and diabetes mellitus presented to the emergency department with complaints of fever, dry cough, and dyspnea for the last week. On initial evaluation, she had tachypnea, tachycardia, a temperature of 100°F, and oxygen saturation of 85% on room air. On auscultation, she had scattered wheezing in both lung fiends. Chest X-ray revealed small infiltrates in the lower lobe of both lungs (Figure 1). A preliminary diagnosis of pneumonia was made, and she was admitted and isolated due to high suspicion of COVID-19. A nasopharyngeal swab for the SARS-CoV-2 polymerase chain reaction (PCR) test was taken. Initial blood investigations were within normal range except for mildly elevated C-reactive protein. Her COVID-19 RT-PCR report was positive, and she was commenced on oral azithromycin 500mg with high-flow supplemental oxygen.

**Figure 2:**
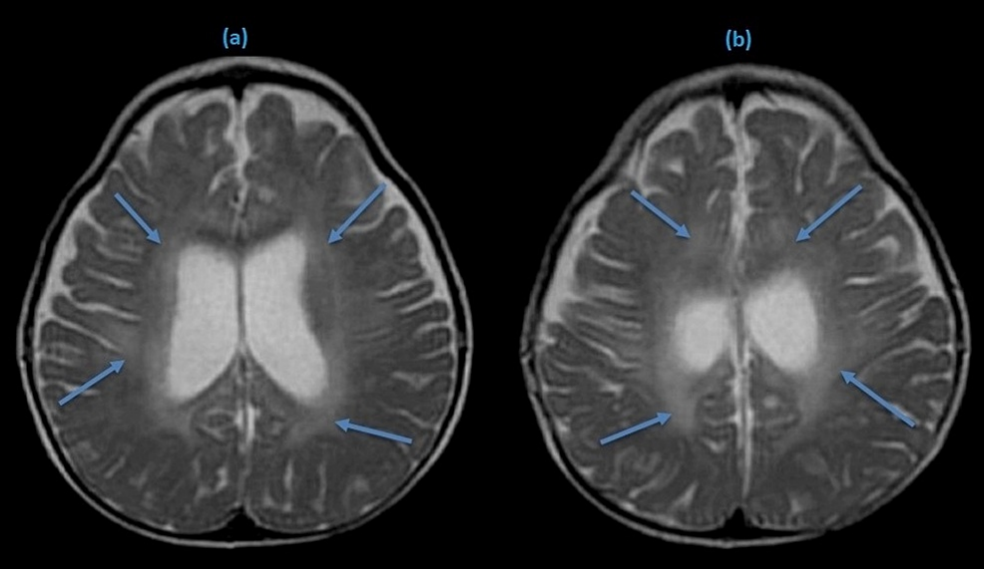
T-2 weighted MRI brain showing hyperintense signals in both hemispheres (blue arrows).

After 17 hours of admission, she developed sudden onset confusion accompanied by restlessness, fearfulness, and visual hallucinations. She was anxious, agitated, and aggressive. Neurological examination revealed an altered level of consciousness, slight tremors of the limbs, and psychomotor restlessness. Her language was inadequate and incoherent, following only simple commands. She also had tremors in her upper extremities without myoclonus or orofacial automatisms. She had no signs of meningeal irritation, neck stiffness, or loss of muscle strength. Computed tomography of the brain was unremarkable, and urgent magnetic resonance imaging (MRI) of the brain was performed, revealing hyperintense signals in frontoparietal and parietotemporal lobes on fluid-attenuated inversion recovery (FLAIR/T2) sequence (Figure 2).

Detailed blood investigations revealed elevated lactate dehydrogenase, creatine phosphokinase, ferritin, and D-dimer. Viral serology was negative for herpes simplex virus (HSV) 1-2, human immunodeficiency virus (HIV), enterovirus, and varicella-zoster (VZV) virus. Blood culture and gram stain did not grow any organism. Her urine screening was negative for any illicit drug use. Her cerebrospinal fluid (CSF) analysis was performed, and the results are shown in Table 1. A diagnosis of acute encephalitis was made due to concurrent infection of COVID-19 and lack of other supporting findings which would suggest a diagnosis other than COVID-19.

**Table 1:** Cerebrospinal fluid (CSF) analysis. VZV: varicella-zoster virus, PCR: polymerase chain reaction, SARS-CoV-2: severe acute respiratory syndrome coronavirus 2, HSV: herpes simplex virus, CMV: cytomegalovirus, VDRL: venereal disease research laboratory.

Spinal fluid	Lab values	Reference range
White blood cells (mm^3^)	2	0-5
Red blood cells (mm^3^)	130	0
Color	Colorless	Colorless
Protein (mg/dL)	66	15-45
Glucose (mg/dL)	81	40-70
Opening pressure (cmH_2_O)	12	05-20
Gram stain	Negative	Negative
VZV PCR	Negative	Negative
SARS-CoV-2 PCR	Negative	Negative
HSV 1-2 PCR	Negative	Negative
CMV PCR	Negative	Negative
VDRL	Nonreactive	Nonreactive
Bacterial antigen	Negative	Negative

Initially, she was treated with intravenous acyclovir and lopinavir in addition to azithromycin orally. However, after the results of CSF analysis, antiviral medications were stopped, she was continued on azithromycin. In addition, intravenous methylprednisolone and tocilizumab were added. Her neurological symptoms started improving gradually after 14 hours. She was responsive, calm, and cooperative after 72 hours of admission. However, a slight postural tremor persisted. She was afebrile on day 5 with no respiratory signs and symptoms. She was discharged on oral azithromycin and dexamethasone with home self-isolation measures on day 12. On her recent visit two weeks later, she was doing well with no neurological signs and symptoms.

## Discussion

Acute encephalitis is a life-threatening condition requiring immediate observation and management. Acute encephalitis is an acute inflammation of the brain and generally presents with fever, and headache, complicated by confusion, seizures, and loss of consciousness [10]. Encephalitis can result from infection (viral, bacterial) or the body’s immune response against the brain tissue. HSV and Epstein-Barr virus are common causes, and encephalitis due to COVID-19 is rarely reported [11,12]. Only a small number of cases have been underlined in the literature. Etemadifar et al. reported a case of acute encephalitis as an initial manifestation of COVID-19 [13]. Moriguchi et al. also highlighted a case of encephalitis associated with COVID-19 [14]. Similarly, Haider et al. reported COVID-19-associated acute encephalitis in an old male [12].

The pathophysiology of acute encephalitis in COVID-19 is not well defined. Angiotensin-converting enzyme 2 (ACE2) receptors are responsible for viral attachment, margination, and internalization. These receptors are expressed in alveolar cells of the lung epithelium, gastrointestinal tract, and the central nervous system, including the hippocampus, midbrain, and glial cells [15,16]. COVID-19 is an inflammatory disease characterized by a widespread immune response throughout the body with the release of inflammatory markers, including cytokines and chemokines. This hyperinflammatory state disrupts the permeability of the blood-brain barrier leading to activation of the neuroinflammation cascade [17,18]. SARS-CoV-2 induces a cytokine storm resulting in immune-mediated damage by molecular mimicry mechanisms such as the production of autoantibodies against the glial or neuronal cells. Another proposed mechanism may be direct damage to the neurons due to severe hypoxia caused by pneumonia, pulmonary edema, and ARDS [15,18].

Acute encephalitis diagnosis is based on clinical presentation, CSF analysis, and brain imaging. However, COVID-19-induced encephalitis is highly challenging to diagnose as the definitive diagnosis of viral encephalitis is based on viral isolation from CSF. Viral isolation is difficult due to transient dissemination of SARS-CoV-2 and low CSF titer [10]. Therefore, the lack of COVID-19 in the CSF sample could not exclude the diagnosis. MRI and computed tomography are usually done to identify the classic changes of encephalitis. COVID-19-induced encephalopathy has shown a broad spectrum of MRI findings such as leptomeningeal enhancement, white matter microhemorrhages, FLAIR signals, and ischemic strokes [19]. Management of COVID-19-induced encephalitis is primarily supportive, and intravenous immunoglobulins, high dose steroids, and immunomodulators have been tried in many cases with variable outcomes [10].

Our case is of particular interest, as the patient presented with a chest infection, followed by neuropsychiatric manifestations. Lack of SARS-CoV-2 in CSF, abnormal findings on MRI, and rapid response to steroids support the benign course of acute encephalitis induced by COVID-19, justifying the potential neurological complication of COVID-19 infection.

## Conclusions

Acute encephalitis is a medical emergency requiring immediate diagnosis and management. Our case highlights the need for urgent evaluation in a COVID-19 patient with neuropsychiatric manifestation for the possibility of acute encephalitis. Due to its variable presentation and involvement of other organ systems, there is a dire need to investigate the neuropsychiatric manifestations prudently as a result of COVID-19, particularly during this ongoing pandemic. Data on the neurological manifestations of COVID-19 is evolving rapidly, and it is pertinent to collect reliable data on short-term and long-term neurological manifestations and complications of COVID-19.
